# Task shifting of point-of-care testing to lay health workers in rural primary health facilities in Nsanje, Malawi

**DOI:** 10.1080/16549716.2026.2721045

**Published:** 2026-09-28

**Authors:** Zibusiso Ndlovu, Rosie Burton, Mieke Steenssens, Kingsley Makwale, Simeon Maleka, Greywilly Madaliso, Gilbert Chapweteka, Fingani Mphande-Nyasulu, Emmanuel Kabwe, Johnny Falconer, Sarah Jane Steele, Tom Ellman, Esther C. Casas, Geoffrey Fatti

**Affiliations:** a Médecins Sans Frontières, Nsanje, Malawi; b Division of Epidemiology and Biostatistics, Department of Global Health, Faculty of Medicine and Health Sciences, Stellenbosch University, Cape Town, South Africa; c Southern African Medical Unit, Médecins Sans Frontières, Cape Town, South Africa; d Ministry of Health, Nsanje District Hospital, Nsanje, Malawi; e Faculty of Medicine, King Mongkut’s Institute of Technology Ladkrabang, Bangkok, Thailand; f Institute of Fundamental Medicine and Biology, Kazan (Volga Region) Federal University, Kazan, Russia

**Keywords:** Lay cadres, point-of-care testing, multi-disease testing, advanced HIV disease, task sharing

## Abstract

**Background:**

Point-of-care testing (POCT) can reduce diagnostic gaps, but because it frequently falls to overstretched frontline healthcare workers (HCW), its real-world effectiveness is diminished. Despite World Health Organization recommendations for task sharing (TS) with lay health workers (LHW), it remains underutilized.

**Objective:**

To explore whether task-shifted POCT by LHWs can improve uptake compared to standard HCW-led care.

**Methods:**

A retrospective study at 5 PHC facilities in Nsanje, Malawi, compared a pre-intervention (model 1) period where HCW conducted POCT (for rapid HIV, AHD, syphilis, hepatitis B and malaria), and post-intervention (model 2) where POCT was shifted to trained LHW. The primary outcome was the total number of POCT performed. The two-sample proportion test was used to assess differences in testing uptake in model 1 and 2.

**Results:**

A total of 3,428 POC tests were conducted by LHWs in model 2 compared to 1,738 in model 1 (a near-2-fold increase). In both models, rapid HIV (38.9%), malaria (21.9%) and CD4 cell count (20.0%) tests were most common. The CD4 cell count testing had the highest incremental change in testing volume of 338% between models (from 192 to 840 tests; *p* < 0.001).

**Conclusions:**

TS for POCT with LHW was feasible in rural PHC facilities and contributed to increased POCT volumes across multiple diseases, particularly for CD4 testing. As the range of POC tests expands, TS can improve uptake of diagnostics in these settings, but it must be accompanied by appropriate training and supportive oversight.

## Paper Context

**Main findings**: Task-shifting point-of-care testing to lay health workers in rural Malawi primary healthcare facilities nearly doubled testing volumes, with CD4 cell count testing showing the greatest incremental increase of 338%.

**Added knowledge**: This study provides empirical evidence that task-shifting point-of-care testing to lay health workers is feasible and effective across multiple disease areas in rural settings, when coupled with appropriate training and supportive oversight.

**Global health impact for policy and action**: National health programs should formalize task shifted point-of-care testing with emphasis on the transversal role of lay health workers for multi-disease testing to expand diagnostic uptake.

## Background

Point-of-care testing (POCT) for disease detection and management is a highly practical and impactful approach, particularly in resource limited settings [1,2]. POCT can enable rapid linkage to care, effective response to disease outbreak and enhance healthcare efficiencies [2–5]. POCT requires minimal infrastructure, and it includes various modalities such as lateral flow assays, handheld devices, near-POC benchtop analyzers among other modalities [6–8]. National essential diagnostic lists (NEDL) in low- and middle-income countries (LMIC) include POC tests among priority in-vitro diagnostic tests crucial for the population’s health needs [9,10]. However, studies suggest that only 19% of the population in LMIC have access to the simplest POC tests for healthy pregnancy and non-communicable diseases (NCDs), excluding HIV and malaria tests [11].

Policymakers, implementers, and funders have largely overlooked POC diagnostics, while shortages of trained testing staff and unreliable supplies have further widened this diagnostic gap [5,12]. Closing the diagnostic gap for six tracer conditions (HIV, hypertension, diabetes mellitus (DM), syphilis, tuberculosis (TB), congenital hepatitis B) has been shown to potentially avert 1.1 million premature deaths in LMICs [11].

Patients typically seek care at primary health care (PHC) facilities, which often lack on-site laboratory services and rely on overburdened frontline healthcare workers (HCWs) for POCT [13]. In these settings, frontline HCW (nurses and clinical staff) often lack flexible time to perform POC tests for HIV, TB, cryptococcal meningitis, malaria, syphilis, hepatitis, cholera, diabetes mellitus among other tests.

Even though significant strides have been made, advancement toward achieving global milestone targets for elimination of AIDS, viral hepatitis, tuberculosis, NCDs (DM, hypertension), and other key health priorities (including syphilis, gonorrhea) remains insufficient and uneven in many regions [14–19]. This is largely due to persistent gaps in screening and diagnosis. With an ever-expanding range of POC tests available, current testing strategies largely reliant on frontline HCW, with limited task sharing to lay cadres [5], are increasingly insufficient. New approaches are needed, especially as national programs face unprecedented funding challenges [20], even while testing demands continue to rise.

WHO endorses task sharing (TS) for POCT with non-laboratory personnel [2,21] to address HCW shortages. TS involves rationally re-distributing tasks from professional HCWs to trained lay health workers (LHWs) [21] through collaboration. LHW are personnel who deliver healthcare-related services but do not possess formal professional or paraprofessional qualifications. Studies have shown that LHWs (counsellors, community health care workers, health diagnostic assistants, phlebotomists, health surveillance assistants) with adequate training can reliably perform POCT services [3,10,22–25]. Nevertheless, other studies note that decentralized TS for POCT demands support from national laboratories and programmes to ensure proper training, supervision and quality assurance [3,26]. Additional research indicates that the implementation of TS for POCT often faces practical hurdles stemming from lack of supportive national policy and funding strategies, intricate governance for POCT conducted outside laboratories and rigid regulations upholding professional task boundaries [5,10].

Although many studies demonstrate that TS of POCT to LHWs is non-inferior to testing by frontline HCW, few have explored the feasibility of task shifted responsibility for multiple POCT with LHW as well as exploring adaptations needed to ensure feasible implementation. Task shifting being the complete allocation of POCT to lay cadres. Such studies could guide national decisions on adoption of task shifted multi-disease POCT and challenge the persistence of rigid professional boundaries together with siloed vertical disease program implementation of task-shifted POCT. The aim of the study was to explore whether task-shifted implementation of several POC tests for different health conditions, utilizing LHWs, could improve uptake of testing, compared to standard-of-care where frontline HCWs were primarily responsible for onsite POCT.

## Methods

### Study background and setting

In 2011, Médecins Sans Frontières/Doctors without borders (MSF) partnered with the Malawi Ministry of Health to improve access to quality HIV/TB care services in Nsanje district, primarily through mentorship and targeted health system strengthening activities. Nsanje is a remote, largely rural district in southernmost Malawi and it is the second poorest district with 74.3% living in poverty [27–29]. Patients face challenges in accessing healthcare due to long distances to health facilities and the flood-prone environment together with socio-economic barriers and this leads to late presentation to care and poor retention. Clinical mentorship, supportive supervision and commodities for the implementation of the AHD package, including differentiated ART delivery models, were provided to Nsanje District Hospital (NDH) and seven other health centres (Sorgin, Kalemba, Phokera, Nyamithuthu, Tengani, Mbenje and Ndamera), in a phased manner as from 2018 [30].

### Study design

This was a retrospective analysis of routinely collected program data from five MSF-supported rural PHC facilities in Nsanje, Malawi. The study compared two models of 6 months each: a pre-intervention period (model 1) where frontline HCW conducted POCT among eligible patients (6 months from July 2018 and Dec 2018), and post-intervention (model 2) where POCT was shifted to five trained LHW (6 months from July 2019 and Dec 2019). Testing data for patients who received POCT for the targeted condition(s) (rapid HIV, syphilis, malaria, CD4 cell count, urine TB LAM and CrAg lateral flow assays) were considered. The study periods were chosen and separated into two models to ensure a clear temporal delineation in capturing POC testing activities. To account for seasonality that may affect specific diseases testing trends, equivalent time intervals before and after deployment of LHWs were chosen for comparison. From the seven PHCs supported by MSF, only five were chosen based on the timeline of MSF support as well as availability of complete and corresponding data that could enable a reliable comparison of models 1 and 2.

### Task shifting intervention for POCT (model 2)

The Malawi department of HIV/AIDS together with non-governmental organizations like Partners-in-Hope deployed health diagnostic assistants (HDAs) to health facilities, to support HIV and syphilis testing services including collection of blood samples for early infant diagnosis (EID), and HIV Viral load (HIV VL), among other roles [31]. HDAs are a lay cadre with a minimum education qualification of a high school certificate and trained for 4-weeks in HIV testing services. Over 1000 HDAs were seconded to over 500 health facilities across all districts of Malawi in year 2015 [32]. From this pre-existing cadre, MSF recruited and provided further training for 3 days on operational procedures for CD4 cell count testing using PIMA instrument (Abbott MA, USA; *formerly Alere*), urine Determine TB LAM test (Abbott MA, USA; *formerly Alere*) and IMMY CrAg lateral flow tests (IMMY Diagnostics, Norman, USA) as well as result documentation, device maintenance and quality control monitoring in POC testing. Training included theoretical and practical segments on sample collection, test performance, result interpretation, biosafety, and confidentiality; following a standardized package previously described [24,31]. Competency was assessed using structured checklists prior to deployment, with all LHWs passing before field deployment [24,25]. The LHWs were each deployed to MSF supported health facilities in Nsanje district and their role was to conduct testing for AHD as well as other POCT (HIV, syphilis, hepatitis B, malaria). The LHWs also conducted other activities including collecting dry blood spot (DBS) samples for HIV viral load (VL) and HIV early infant diagnosis (EID). Ongoing performance evaluation and supportive supervision were regularly provided to LHWs by the MSF district laboratory manager, and this included monthly site audits, regular QC record review, annual competency re-assessment, proficiency testing, and troubleshooting support to ensure quality assurance (QA) in POCT. Testing consumables were supplied for POC tests.

At each PHC, a dedicated ‘POC room’ was set-up and equipped with POC tests as well as a PIMA CD4 device and it was often in direct proximity to the outpatient section to facilitate prompt results return. Patients eligible for testing were identified in the triage and sent to the POC room and thereafter returned to the consultation room for further clinical management. The objective was to return all testing results to the clinicians within 30–60 minutes from sample venesection to allow for quick diagnosis and further management decisions in the same clinical encounter. MSF clinical staff supported and mentored national clinical staff for same-day result review and patient management. Details of the intervention in PHCs can be found elsewhere [30].

In the POC room, the performance of the PIMA CD4 analyzer was monitored daily using standard PIMA beads as part of the quality assurance (QA) process. Additionally, left-over patient samples at Nsanje district hospital (NDH) laboratory were randomly sent to POC room in the PHCs for testing in the PIMA CD4 analyzer to facilitate inter-laboratory comparison and quality verification.

### Baseline testing services before POCT task shifting intervention (model 1)

Prior to the intervention, basic POC tests were conducted within the PHCs mainly by frontline HCW (nurses) sometimes with support of HIV testing counsellors and health surveillance assistants. Samples for AHD testing were sent through an ambulance (if available) to the district hospital laboratory (Nsanje district hospital, NDH) for testing, Table 2. For AHD, all EDTA blood samples for CD4 testing were reflex tested at NDH laboratory for CrAg if CD4 was less than 200 cells per cubic millimeter (CD4 < 200cells/mm^3^). Frontline HCW also collected a DBS for HIV VL and HIV EID among eligible patients. Testing results were received a few days or weeks later, but in anticipation of the delays, nurses told patients that the results would be available at the next scheduled appointment (the majority scheduled 4 weeks later).

### Study hypotheses

We hypothesized that task-shifted implementation of multi-disease POC tests, utilizing LHWs, could improve testing uptake among eligible patients, compared to standard-of-care where frontline HCW were primarily responsible for onsite POCT.

We expected that if the task-shifted POCT program was effective, there would be higher pre-to-post differences in POC testing volumes. By implication, this could show that the task-shifted POCT program had contributed to higher POCT at the clinic level.

### Study outcomes

- The primary study outcome was the testing uptake, defined as the number of different POCT conducted in model 1 compared to model 2, in time-period.
- Secondary outcomes, for model 2, included;
  - The proportion of patients retained at each step of AHD cascade, after an initial CD4 < 200cells/mm^3^ together with subsequent urine TB LAM and CrAg POC tests. The cascade of testing outcomes was included because it can help to demonstrate the testing uptake of sequential AHD POC testing steps that patients undergo, and this can identify gaps in task shifted testing at each stage.
  - The CD4 testing coverage achieved in model 2, defined as the proportion of patients receiving a CD4 cell count test among eligible patients (those initiating ART and those with high HIV VL).
  - The total PIMA CD4 instrument error rate in the healthcare facilities in model 2.

### Data management and analysis

For model 1, data officers abstracted clinic aggregate anonymized POCT data for patients tested during the time-period. Data was abstracted from the hard copy ‘testing and sample management registers’ within consultation rooms. Abstracted data included; aggregate data for all POCT tests conducted and details of samples referred to NDH laboratory (HIV VL, HIV EID, CD4). For model 2, data was abstracted from POCT registers in POC room. Abstracted data included individual POCT tests and the results as well as the reason for testing (if AHD screening) and data for other samples referred to NDH laboratory (HIV VL and HIV EID). The information was transferred to an Excel spreadsheetand cross-referenced with hard copy registers for verification and completeness. AHD testing data for model 1 was also confirmed at NDH lab database. The study analysis focused on the number of tests conducted (testing uptake) across different diseases as the main outcome, especially as patient-level differentiation was impossible from model 1 data. Testing uptake was considered a proxy measure for program activity as it can reflect changes in utilization of testing services.

Unique alphanumeric codes were used to identify individual patients in model 2 data, and no patient identifiers were included in the analysis database. The data was imported to STATA 16.0 for analysis.

Descriptive statistics were used to summarize testing volumes, patient characteristics and crude summaries of the AHD testing cascade. Absolute numbers and proportions were used to describe categorical variables while median and interquartile range (IQR) were used for continuous variables. Disaggregated data from model 1 was unavailable for further comparison. The two-sample test for proportions was used to test for differences in the models.

The study was approved by the Malawi National Health Sciences Research Committee (21/08/2759), the Stellenbosch University Health Research Ethics Committee (S23/11/298) and by the MSF ethics review board. The study was carried out in accordance with the principles of the Declaration of Helsinki. Written informed consent was waived as data were collected retrospectively and all clinical elements (POC and laboratory-based testing) followed standard of care practice.

## Results

### Testing conducted in the models

Between June 2018 to Dec 2018, a total of 1,738 POC tests were conducted within the 5 health facilities for model 1 whereas 3,428 were conducted for model 2 between June 2019 - Dec 2019, Tables 1 and 2. In both models, the majority of the tests conducted were for HIV rapid test (38.9%), malaria (21.9%) and CD4 cell count (20.0%) (Tables 2 and 3). The CD4 cell count testing had the highest incremental change in testing volume of 338% between model 1 (192 tests) and 2 (840 tests) and this difference was statistically significant (*p* < 0.001).

**Table 1.** Size of the active ART cohort in the health facilities considered for the study.

Health facility name	Ndamera health centre	Sorgin health centre	Mbenje health centre	Nyamithuthu health centre	Tengani health centre
Active ART cohort size (Dec 2019)	2075	1504	1365	543	1485
Active ART cohort size (Dec 2018)	1763	1252	1197	447	1317

Note: the size of the active cohort was estimated from data at the end of the intervention periods (for model 1, it was December 2018 and for model 2 it was December 2019).

**Table 2.** Number of tests conducted at each PHC in model 1 (July 2018 to Dec 2018) and model 2 (July 2019 to Dec 2019).

Health facility		Ndamera			Mbenje			Sorgin			Nyamithuthu			Tengani ‡		
Model for POC and-or laboratory testing		Model 1	Model 2	% change	Model 1	Model 2	% change	Model 1	Model 2	% change	Model 1	Model 2	% change	Model 1	Model 2	% change
Tests done	HIV rapid test	168	258	+54%	132	204	+55%	279	386	+38%	90	134	+49%	112	164	+46%
	Malaria rapid test	154	188	+22%	48	78	+63%	130	197	+52%	52	86	+65%	84	114	+36%
	Syphilis rapid test	48	71	+48%	32	51	+59%	68	94	+38%	28	39	+39%	38	67	+76%
	Advanced HIV disease • CD4 • LAM • CrAg	46* †* †*	195 42 31	+324%	50* 14* †*	155 36 35	+210% +157%	50* 5* †*	228 46 48	+356% +820%	17* †* †*	66 14 11	+288%	29* †* †*	196 59 34	+810%
	Samples referred to NDH lab for: • EID testing • HIV VL testing	70 185	101 366	+237% +98%	34 56	65 102	+171% +82%	43 113	73 140	+70% +24%	9 41	25 68	+178% +66%	39 107	80 139	+101% +30%
Distance from health facility to NDH laboratory		24 km			13 km			60 km			33 km			22 km		

Key: %change- percentage difference between models; † this information could not be found; *tests were referred to NDH lab; ‡ MSF support activities commenced in September 2019.

**Table 3.** Total number of tests conducted in each of the models.

POC tests done on site	Model 1	Model 2	% change	*p-value* †
**Overall total**	1738	3428	+97%	‡
HIV rapid test*	821	1171	+43%	‡
Malaria rapid test*	468	661	+41%	‡
Syphilis rapid test*	224	400	+79%	‡
AHD screening				
• CD4 testing	192	840	+338%	<0.001
• Urine TB LAM	33	197	+499%	<0.001
• CrAg	*	159		

Key: †two-sample test for proportions; ‡the denominator (total eligible population) was unknown and statistical tests for significance between the two models could not be performed; *this information could not be found.

### Comparison of testing models

Overall, there was a near 2-fold increase (97%) in proportion of tests conducted within the PHCs during the model 2 period (3,428) compared to model 1 (1,738) (Table 3). POC tests for CD4 cell count had the highest percentage changes of 338% between the two models. To enable statistical comparison between the two models using the two-sample proportion test, data on total eligible patients among all the POC tests conducted was necessary, however, it could only be accessed for CD4 testing among patients initiating ART and those with high HIV VL. As such, for CD4 cell count, the testing volumes between the 2 models were significantly different (*p* < 0.001) (Table 3). Further characteristics of patients tested for AHD are described (Table 5).

Model 2 was linked with an increase in the proportion of HIV EID and HIV VL samples referred to centralized molecular laboratory for testing, by 77% and 62% respectively (Table 4).

**Table 4.** Total number of HIV EID and HIV VL tests referred to NDH in each of the models.

Total samples referred to NDH	Model 1	Model 2	*% change*	*p-value*
HIV EID	195	344	+77%	‡
HIV VL	502	815	+62%	‡

Key: ‡the denominator (total eligible population) was unknown and statistical tests for significance between the two models could not be performed.

### Secondary study outcomes

#### AHD testing cascade for PHCs facilities in model 2

Between July 2019 and Dec 2019, lay cadres conducted POC CD4 testing for a total of 840 patients at 5 PHC facilities (Table 5). Based on patients newly initiating ART (538) and those with unsuppressed HIV VL (88) during model 2 time-period, this represented an estimated 86% (626/724) CD4 cell count testing coverage. Among patients with recorded characteristics, an estimated 53% of the patients were female and the median (IQR) age was 38 [30 – 46] years. A total of 164/840 (19.5%) patients had CD4 < 200cells/mm^3^. A total of 15/197 (8.6%) tested positive for urine TB LAM whereas 8/159 (5%) tested positive for plasma CrAg (Table 5).

**Table 5.** Characteristics of patients screened for AHD in the 5 health facilities (in model 2).

Variable	Total tested; (%)
Tested for CD4 cell count per facility	
Ndamera	195 (23.2)
Mbenje	155 (18.5)
Nyamithuthu	66 (7.9)
Sorgin	228 (27.1)
Tengani	196 (23.3)
Reason for CD4 testing*	
ART commencement	538 (64.0)
Catch-up/baseline CD4‡	20 (2.4)
High HIV VL (>1000copies/ml)	88 (10.5)
Stage 3 or 4	45 (5.4)
Return after LTFU	6 (0.7)
Unknown†	143 (17.0)
%CD4 < 200 cells/µl	164/840 (19.5)
% Urine TB LAM positive	15/197 (8.6)
% CrAg positive	8/159 (5.0)

Key: *Reason for CD4 testing is not mutually exclusive; **‡**Catch-up CD4 cell count test for all without ever documented CD4 test prior; **†**reason for CD4 cell count testing was not systematically captured.

Figure 1 below shows the successive steps of the AHD testing cascade at the PHCs. Among 164 patients with CD4 < 200cells/mm^3^ at PHCs, there was a high uptake of urine TB LAM (197 tests with 8.6% positive) and serum CrAg (159 tests with 5.0% positive).

**Figure 1.**
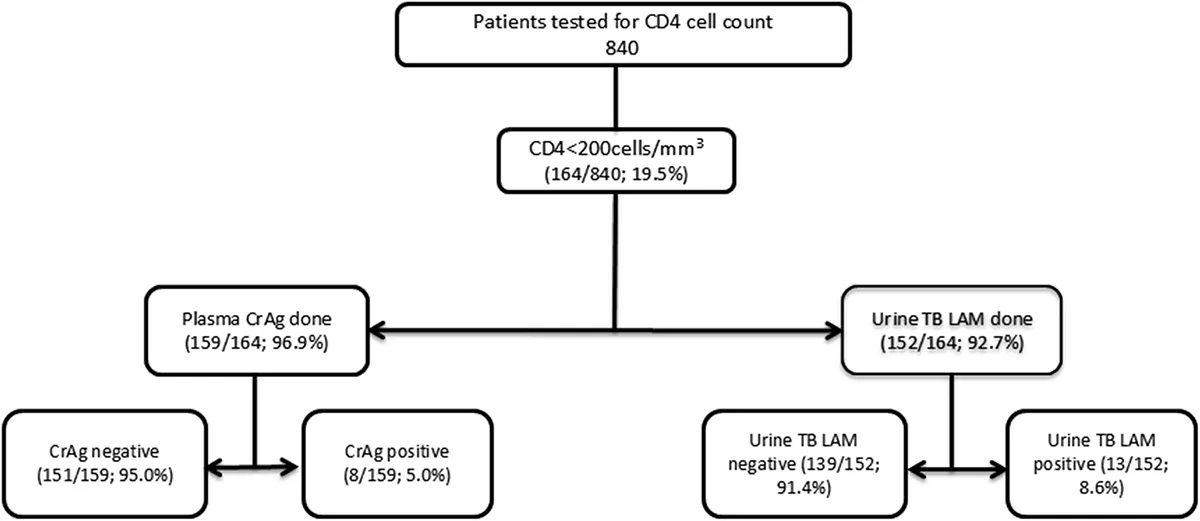
Overall cascade of AHD POC testing in 5 PHC for patients with CD4 less than 200cells/mm3.

Across all health facilities, the total PIMA CD4 error rate was 8.3% (70/840). The leading causes were invalid test error code 860 (36.8%), followed by error code 201 (18.7%) and error code 940 (12.9%), which together accounted for almost 70% of all errors. According to manufacturer recommendations, error code 860 typically stems from equipment or reagent-related issues whereas error codes 940 and 201 can be either operator or equipment-related [31].

## Discussion

This study demonstrates that a task-shifting model to LHWs for the implementation of POC testing for multiple health conditions is feasible at PHC levels and it was linked with significantly increased POC testing volumes compared to before, when POC testing was the responsibility of frontline HCWs.

Task-shifted POC testing was linked to a near-two-fold increase in the POC tests conducted in model 2 compared to model 1 as well as associated with a significantly increased proportion of samples referred to the district laboratory for molecular testing. By delegating POC testing responsibilities, frontline HCW can free up valuable time, allowing them to attend to a larger number of patients per clinic session. This redistribution of workload can optimize clinic efficiency and enhance access to essential diagnostic services. This aligns with other research studies as well as WHO recommendations which have emphasized that LHW are well placed to perform POC testing [5,10,21,23,24], compared to frontline HCW who have less availability for additional tasks. Prior research also highlights that the greatest benefits from task shifting could be more pronounced in PHC care settings [5,33] as task shifting can significantly expand access to timely diagnostics and reduce unnecessary referrals of patients or samples to higher-level health facilities. This could save the patient time and cost as well as potentially contribute to improving global health milestones, as echoed by other studies [25,31,34]. In many settings, there are existing lay cadres whose scope could be broadened/re-modelled to include task shifted testing for POC tests [5,10]. With decreased international funding, enhancing integrated diagnostic testing efficiencies, amongst diminishing resources, is a priority for low- and middle-income countries.

In model 2, task shifted CD4 POC testing contributed to a moderately high CD4 cell count testing coverage of 86% among patients initiating ART and or with high HIV VL. This could support early identification of patients with AHD, especially as measurement of CD4 has declined to very low levels since the treat-all recommendation, more-so in sub-Saharan Africa [35].

Even though some studies have highlighted that implementation of POC tests could inevitably increase patient waiting times and or cause patients to exit care while waiting between rooms [36], for this study the POC room became an essential part of the patient flow with patients progressing through the various clinic stations (waiting room, clinician consultation, POC-room, and pharmacy). The POC room provided a defined space for POC testing, and this could improve the performance of tests within controlled environments and reduce the variability introduced by busy-complex clinical areas, which usually lack work surfaces for the array of POC tests. However, insufficient documentation of POC test result turn-around time and subsequent communication of results to clinicians in our dataset prevented us from directly commenting on this.

Effective user training and ongoing monitoring support are crucial for task shifted POCT services. For this study, the district laboratory manager supported and mentored the LHWs in the five PHCs for POCT quality, to help maintain the trueness results. The 8.3% PIMA CD4+ cell count testing error rate obtained in our study is comparable with error rates from a systematic review study that found PIMA CD4 median error rates of 8–10%, depending on the setting [37]. Such results are indicative of the acceptable quality of task shifted CD4 testing services.

The production of the PIMA CD4 instrument was discontinued in March 2022, by the manufacturer (Abbott, Jena, Germany) due to the low volumes of CD4 tests ordered [31]. This has resulted in a considerable reduction in available CD4 testing options, and access to CD4 testing has become a substantial barrier for the AHD package of care [8].

While task shifting holds great promise, it must be implemented with clarity on LHW work-roles along with POCT, otherwise it can lead to ‘task dumping’ with potential deterioration in POCT quality. To support sustainable scaling of TS for the repertoire of POC tests with LHW, especially in decentralized health facilities, national health policy and strategy reforms are needed to support formalization of task shifted multi-disease POCT. Recent reports highlight that the HDA lay cadre was phased-out in Malawi as they had no formal technical training and that the Medical Council of Malawi did not recognize or register them [38]. The report further highlights that this process was undertaken to standardize the numerous lay cadre titles originally created by various non-governmental organizations, thereby preparing for their systematic transition into government health services. Nevertheless, long-term sustainability of TS for POCT services could be assured if national laboratory policies and strategies as well as vertical disease implementation strategies emphasize the transversal role of LHW for multi-disease POCT [10]. Establishing clear guidelines on POC tests suitable for task-shifting to LHW, alongside national training programs for POCT and a framework for sustained support of delegated testing, is essential.

The study strengths include the use of programmatic data from decentralized rural PHCs, in a high HIV prevalence setting. Limitations of the study include the fact that the retrospective methodology was based on routinely collected program data, which was of suboptimal quality and not complete and this limited study data analysis and generalizability of findings. In model 1 (pre-intervention), unique patient-level differentiation with tests (distinct patients and repeat tests) was impossible and this could have contributed to overinterpretation. However, patient-level testing data of model 2 provided contextual checks (and confidence in interpretation of uptake and coverage) to model 1 data, thereby strengthening the reliability of the study findings. The study relied on test counts as the primary outcome as test volume was considered a pragmatic measure of scale and reach, under real-world constraints. Inconsistent POC test kit availability may have affected testing volumes, especially in pre-intervention period (model 1) and some of the increase in testing uptake in model 2 could possibly be attributed to more consistent access to tests. This could have contributed to temporal confounding. Fewer CD4 tests requested in the pre-intervention period could have been because the AHD package of care was still novel as initial WHO guidance was introduced in 2017 and Malawi had just commenced its adoption. Equally, the health facility program maturation and experience among health care workers could have contributed to more patient testing volumes. Due to poor documentation, the CD4 testing coverage in this study was estimated only among patients newly initiated on ART and patients with unsuppressed HIV VL and we could not include patients who were non-adherent to ART or returning after loss-to-follow up or with clinical signs and symptoms of AHD. As such, our estimate for CD4 coverage could be biased, although the bias is likely minimal as eligibility ‘reasons for CD4 testing’ are not usually mutually exclusive.

Given the retrospective study design with inaccessible denominators (particularly for model 1) and aggregate count data only, the two-sample proportion test was conservatively adequate for comparing the primary outcome of total POC tests performed between the two independent groups, with sufficient statistical power from our large sample sizes (3,428 and 1,738 tests). While more robust methods like Poisson regression or interrupted time-series analysis would better account for facility-level clustering and secular trends, the two-sample proportion test provided a feasible and statistically valid approach that reduced the risk of overfitting from complex models with incomplete data.

Facilities supported at different intervention start times and only for a short period (3 months) before MSF exit were excluded, to reduce the risk of potential secular changes in testing trends. Furthermore, the study took place in MSF supported health facilities, which are usually better resourced, which could be a challenge for some national programs.

Nonetheless, with the expanding range of POC tests, further research could explore differentiated adaptations in task shifted testing workflows as well as explore qualitative perceptions and acceptance of task shifted POCT. Other studies on time motion may be necessary to evaluate patient flow and waiting times for POCT and management, to determine and improve overall PHC patient flows.

## Conclusion

Our findings indicate that task shifting for POCT for numerous health conditions, utilizing LHWs, is feasible at PHCs and it was associated with increased testing volumes, particularly for CD4 cell count. Lay cadre led POCT could improve the uptake of the repertoire of POC tests; a critical advantage as national health programs confront unprecedented funding challenges. Lay testing should be viewed as part of health systems strengthening, and national laboratory leadership should lead in setting up a framework for supporting lay testers in PHCs.

## Data Availability

The datasets will be made available (DOI link) after the manuscript is accepted for publication.
